# Jejunal Perforation Due to Internal Pancreatic Stents Following Pancreaticoduodenectomy: A Case Series and Technical Modification

**DOI:** 10.7759/cureus.77992

**Published:** 2025-01-26

**Authors:** Shunsuke Tamura, Shinichiro Irabu, Hirotaka Yamamoto

**Affiliations:** 1 Hepato-Biliary-Pancreatic Surgery, Seirei Hamamatsu General Hospital, Shizuoka, JPN

**Keywords:** internal stent, jejunal perforation, pancreaticoduodenectomy, pancreatic stent, postoperative complication, surgical technique

## Abstract

Pancreaticoduodenectomy (PD) is a complex surgical procedure performed to treat various conditions, including pancreatic head cancer and ampullary tumors. Postoperative pancreatic fistulas (POPFs) remain one of the most challenging complications of PD, with potentially severe consequences such as abscess formation, bleeding, and even mortality. To mitigate the risk of POPFs, internal pancreatic stents are often employed to promote healing by diverting pancreatic secretions. Although internal stents offer advantages, including the avoidance of external drainage, complications such as stent migration, cholangitis, and liver abscesses have been commonly documented. A jejunal perforation caused by internal pancreatic stents, however, is an extremely rare complication. In this report, we describe three cases of jejunal perforations caused by internal pancreatic stents following PD and present a modified technique to reduce the risk of this complication.

Three cases of jejunal perforation following PD were observed. In Case 1, a 69-year-old female developed intra-abdominal abscesses, and computed tomography (CT) revealed a perforation caused by a pancreatic stent, necessitating reoperation. In Case 2, an 83-year-old male exhibited a perforation and hematoma on CT 36 hours after surgery, requiring reoperation. In Case 3, a 66-year-old male presented with an inflammatory response five days post-surgery. CT revealed stent-related perforation, leading to reoperation. All patients fully recovered following surgical intervention.

These cases underscore the importance of careful pancreatic stent placement during PD to minimize complications. The likely cause of the jejunal perforations was the perpendicular angle of the stent placement, leading to erosion of the jejunal wall. To address this issue, a modified technique was introduced, involving advancing the stent 5 cm into the jejunal lumen to reduce any direct pressure on the jejunal wall. This adjustment has prevented further cases of perforation, highlighting the critical need for surgical vigilance and technical modifications to enhance patient outcomes following PD.

## Introduction

Pancreaticoduodenectomy (PD) is a complex surgical procedure performed for various benign and malignant conditions affecting the periampullary region, including pancreatic head cancer, duodenal cancer, and ampullary tumors. Despite improvements in surgical techniques and perioperative management that have led to reduced mortality rates of less than 5% in high-volume centers, PD is still associated with significant postoperative morbidity [1,2]. Among the most common and challenging complications following PD are postoperative pancreatic fistulas (POPFs), which can result in serious consequences such as intra-abdominal abscesses, bleeding pseudoaneurysms, and, in severe cases, postoperative mortality due to the enzymatic activity of pancreatic secretions [1-3].

To mitigate the risk of a POPF, various strategies have been employed, one of which is the use of a pancreatic stent across the pancreatojejunostomy [4]. The stent helps to maintain duct patency and promotes the healing of the anastomosis by diverting pancreatic secretions [5]. Both internal and external stenting have been utilized in clinical practice, with no significant differences reported in terms of POPF incidence or patient quality of life [6-8]. Internal stenting, however, is often preferred due to the elimination of external drains, which may cause patient discomfort and complications related to prolonged external drainage [7,9,10]. Despite its advantages, internal stenting is not without risks. Abnormal stent migration has been documented in previous studies, with complications such as cholangitis, liver abscesses, and pancreatitis reported in some cases [11-14]. However, jejunal perforation caused by internal pancreatic stents is an exceedingly rare complication, and there are a few reports of this occurrence in the literature [15-17].

In this report, we present three cases of jejunal perforations caused by internal pancreatic stents following PD. These cases highlight the potential complications associated with internal pancreatic stents and emphasize the importance of careful consideration in stent placement techniques to minimize the risk of perforation. Furthermore, we describe a modification in the stent placement technique that has been implemented to prevent future occurrences of stent-related jejunal perforation.

## Case presentation

Case 1

**Figure 1:**
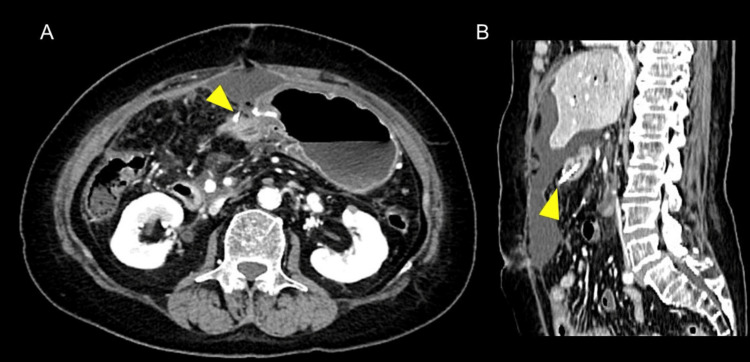
Enhanced abdominal computed tomography of Case 1. Computed tomography showed the pancreatic stent penetrating the jejunum (arrowhead). A: axial view, B: sagittal view.

A 69-year-old female with a history of fatty liver underwent PD for pancreatic head cancer. An end-to-side, duct-to-mucosa pancreatojejunostomy was performed with a trans anastomotic 6Fr internal pancreatic stent (MD-41520, SB-KAWASUMI, Kanagawa Japan), secured with rapidly absorbable chromic sutures. Postoperatively, the patient developed intra-abdominal abscesses, which required repeated drainage. On postoperative day 36, computed tomography (CT) revealed that the pancreatic stent had perforated the jejunal wall, opposite the site of the pancreatojejunostomy (Figures 1A, 1B).

**Figure 2:**
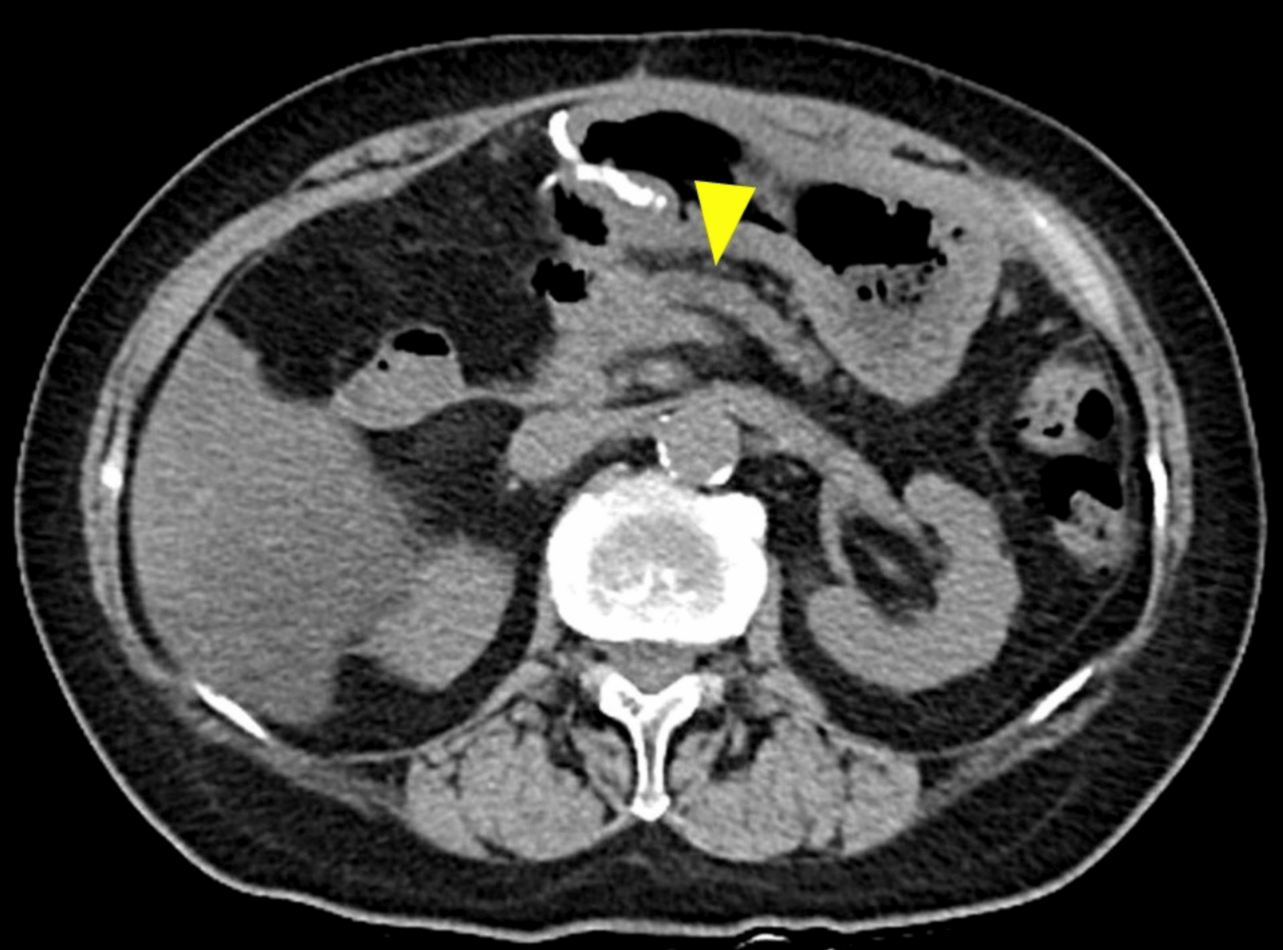
Plain abdominal computed tomography of Case 1 performed four months after the initial surgery. Computed tomography showed no pancreatic stent and no evidence of intra-abdominal fluid collection (arrrowhead: pancreas).

An emergency reoperation was performed. The greater omentum covering the perforation in the jejunum was excised, and the pancreatic stent, which had perforated from the jejunum into the abdominal cavity, was identified and removed. The perforation in the jejunum was sutured closed. The patient's vital signs stabilized from the first day after reoperation, and no abdominal pain was observed. The patient was discharged 54 days after the initial surgery, and postoperative CT revealed no abnormalities (Figure 2).

Case 2

**Figure 3:**
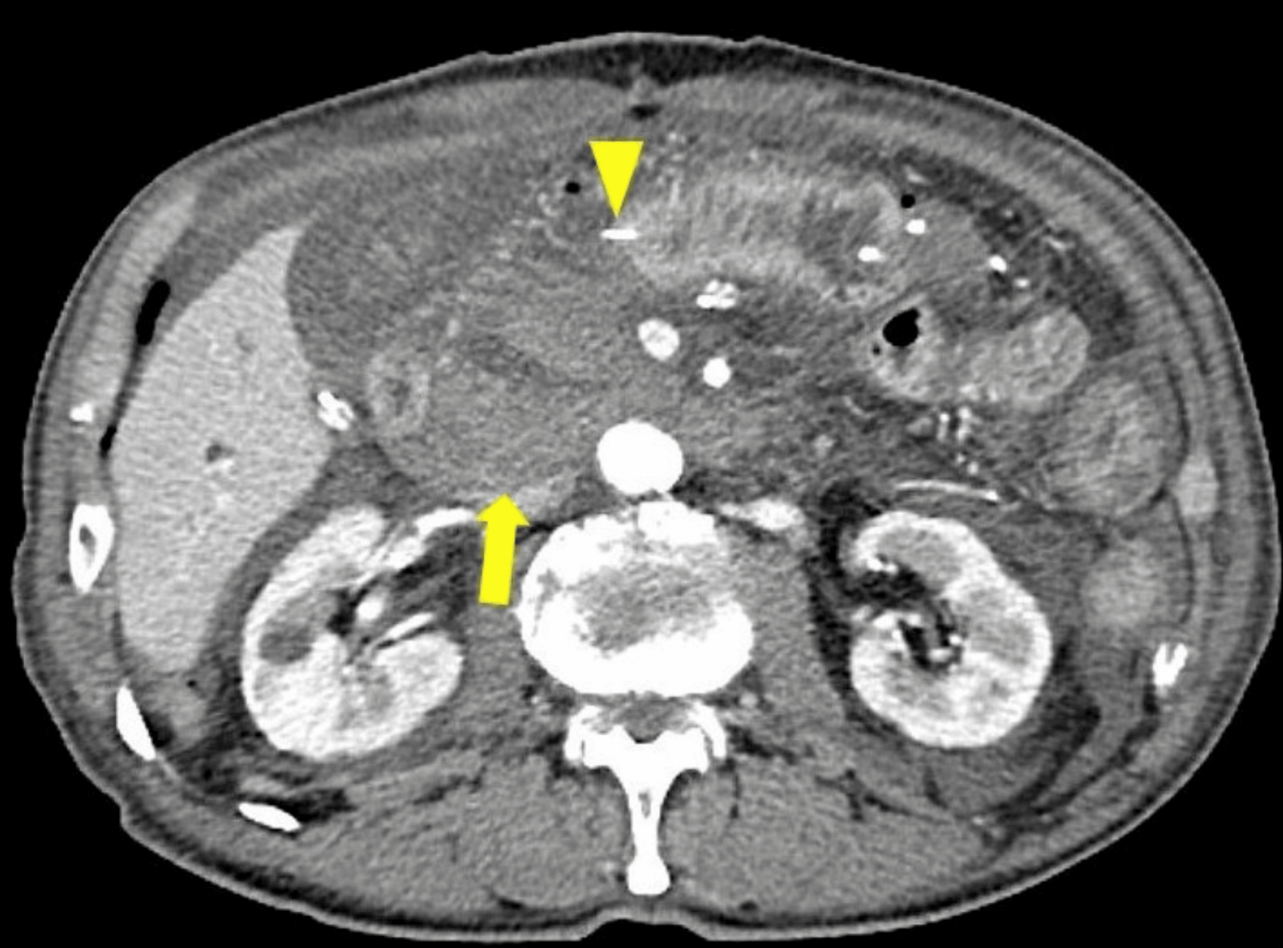
Enhanced abdominal computed tomography of Case 2. Computed tomography showed the pancreatic stent penetrating the jejunum (arrowhead) and a hematoma on the dorsal section of the jejunal loop (arrow).

An 83-year-old male with a history of atrial fibrillation underwent PD for pancreatic head cancer. An end-to-side, duct-to-mucosa pancreatojejunostomy was performed with a trans anastomotic 5Fr internal pancreatic stent (MD-41515, SB-KAWASUMI, Kanagawa Japan), secured with rapidly absorbable chromic sutures. Bleeding from the drain was observed 36 hours after the initial surgery. Emergency CT revealed that the pancreatic stent had penetrated the jejunal wall, contralateral to the pancreatojejunostomy, and a hematoma was noted dorsal to the jejunal loop (Figure 3). 

**Figure 4:**
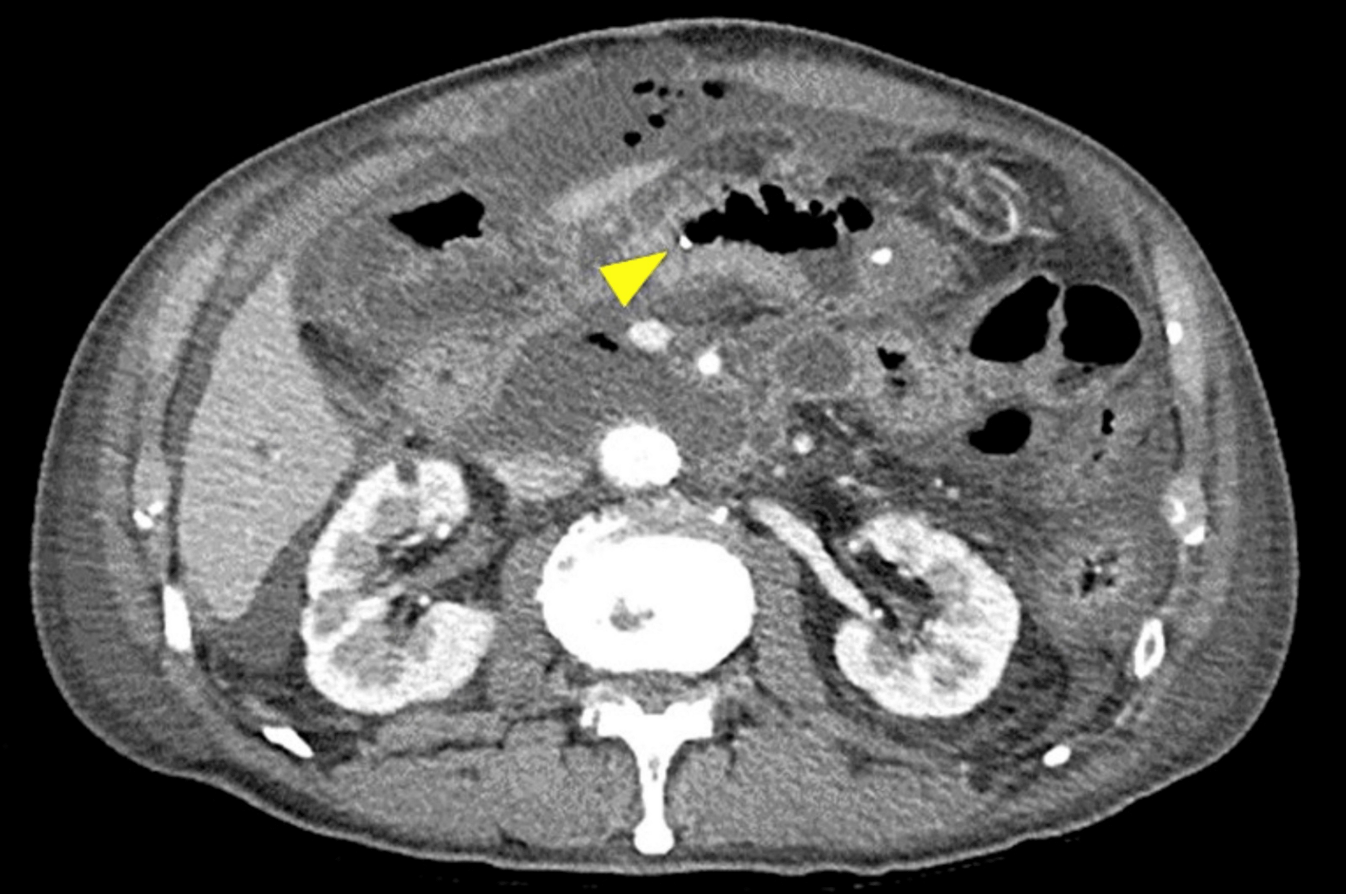
Enhanced abdominal computed tomography of Case 2 performed 11 days after the reoperation. Computed tomography showed the distal end of the pancreatic stent located within the jejunal loop (arrowhead).

An emergency reoperation was performed. The pancreatic stent had perforated the jejunal wall and damaged a mesenteric vessel, causing the bleeding. The mesentery of the jejunal stump was divided, the pancreatic stent penetrating the jejunal wall was resected, and the perforation in the jejunal wall was sutured closed. The patient's vital signs stabilized from the first day after reoperation, with no abdominal pain reported. Postoperative CT revealed no abnormalities (Figure 4). The patient was discharged 51 days after the initial surgery.

Case 3

**Figure 5:**
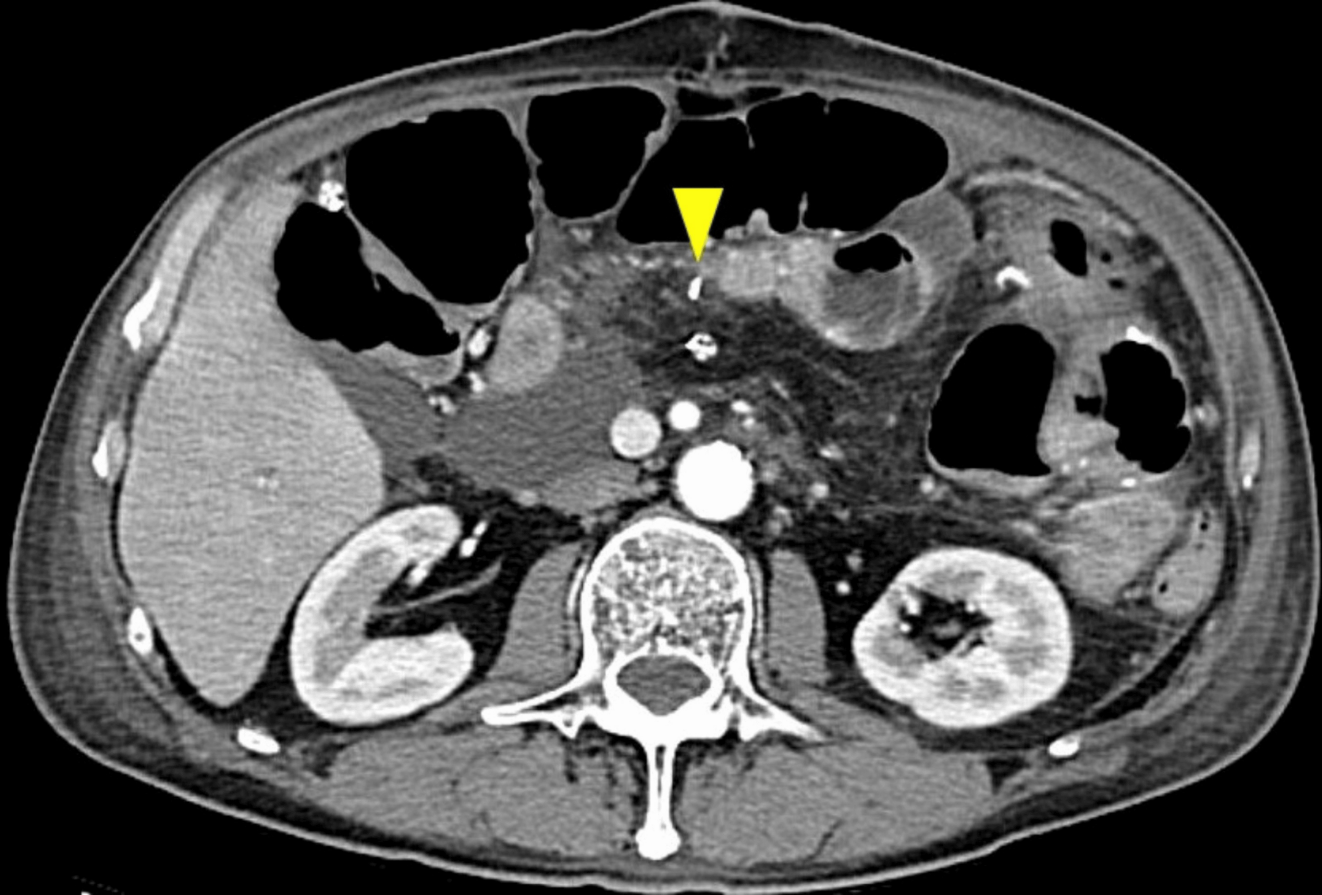
Enhanced abdominal computed tomography of Case 3. Computed tomography showed the pancreatic stent penetrating the jejunum (arrowhead).

A 66-year-old male with a history of hypertension underwent PD for duodenal cancer. An end-to-side, duct-to-mucosa pancreatojejunostomy was performed with a trans anastomotic 6Fr internal pancreatic stent (MD-41520, SB-KAWASUMI, Kanagawa Japan), secured with rapidly absorbable chromic sutures. On postoperative day five, blood tests revealed an increased inflammatory response. An emergency CT scan showed that the pancreatic stent had penetrated the jejunal wall, contralateral to the pancreatojejunostomy, prompting an emergency reoperation (Figure 5).

**Figure 6:**
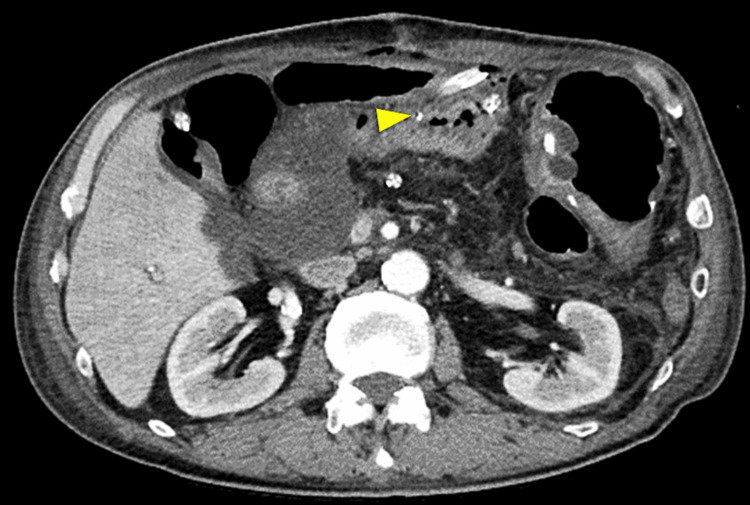
Enhanced abdominal computed tomography of Case 3 performed 5 days after the reoperation. Computed tomography showed the distal end of the pancreatic stent located within the jejunal loop (arrowhead).

The mesentery of the jejunal stump was divided, the pancreatic stent penetrating the jejunal wall was resected, and the perforation in the jejunal wall was sutured. The patient's vital signs stabilized from the first day after reoperation, with no abdominal pain reported. Postoperative CT revealed no abnormalities (Figure 6). The patient was discharged 37 days after the initial surgery.

Surgical procedure

**Figure 7:**
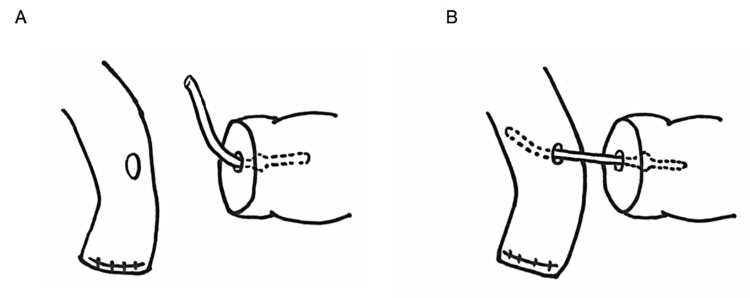
Technique for internal pancreatic stent placement. A) A pancreatic stent, cut to about 6 cm, was inserted into the main pancreatic duct; B) The opposite end of the stent was introduced through the anastomotic opening into the jejunum. Image Credits: Shunsuke Tamura

In the cases prior to the jejunal perforation incidents, the internal pancreatic stent was initially managed by cutting the stent to a length of approximately 6 cm. The pancreatic stent was first inserted into the main pancreatic duct, and the opposite end of the stent was then introduced through the anastomotic opening into the jejunum. The stent was secured in place using absorbable sutures (Figures 7A, 7B).

**Figure 8:**
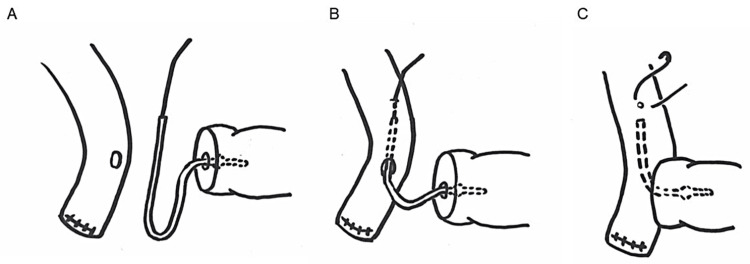
Technique for internal pancreatic stent placement. A) A pancreatic stent was inserted into the main pancreatic duct; B) The aluminum needle attached to the stent was introduced through the anastomotic opening into the jejunum, advanced 5 cm distally, and passed through the jejunal wall; C) The stent was secured with absorbable sutures and trimmed, and the perforation site in the jejunal wall was closed with sutures. Image credits: Shunsuke Tamura.

Following the occurrence of a jejunal perforation in several cases, we modified our technique. In the revised approach, the pancreatic stent was first inserted into the main pancreatic duct and was secured with absorbable sutures. Subsequently, the aluminum needle attached to the stent was introduced through the anastomotic opening into the jejunum and advanced approximately 5 cm distally along the jejunal lumen toward the anal side. The stent was then passed through the jejunal wall and trimmed, and the area where the stent perforated the jejunal wall was closed with sutures (Figures 8A-8C).

## Discussion

The placement of a pancreatic stent across the pancreatojejunostomy is a widely accepted strategy to reduce the risk of a POPF after PD [4,5]. However, with the increasing use of pancreatic stents, complications have also been reported [11-14]. For external pancreatic stents, complications such as unintentional dislodgement of the stent or pancreatic duct strictures after removal have been noted [7,17]. On the other hand, in the case of internal pancreatic stents, complications such as cholangitis, pancreatitis, and liver abscesses due to stent migration have been observed, with pancreatic stent-related complications often occurring within 1-2 years postoperatively [12]. There have been two case reports of small bowel perforations caused by migration of internal pancreatic stents, occurring 2 and 19 years postoperatively [15,16]. However, cases of an early postoperative jejunal perforation on the contralateral side of the pancreatojejunostomy, as seen in our cases, are exceedingly rare, with only one previously reported case in the literature [17]. In our report, we present three cases in which the trans anastomotic stent perforated the jejunal wall contralateral to the pancreatojejunostomy, leading to significant postoperative complications, including an intra-abdominal abscess, hematoma, and perforation requiring surgical intervention.

The jejunal perforation observed in these cases may be attributed to the angle of the pancreatic stent insertion, which likely contributed to the stent's perforation. Initially, the stents were inserted with a shorter length, causing direct contact with the jejunal wall at a near-perpendicular angle. In a previous case report, it was noted that the angle at which the pancreatic stent contacted the jejunal wall was nearly perpendicular, leading to irritation of the jejunal wall and resulting in perforation [17]. Similarly, the stent was placed at a nearly perpendicular angle to the jejunal wall in our present cases, thus causing mechanical stress at the point of contact, leading to the eventual erosion and penetration of the jejunal wall.

These cases highlight the need for careful consideration of the pancreatic stent placement technique during pancreatojejunostomy. To prevent similar complications, it is important to insert the stent at a less perpendicular trajectory, avoiding direct pressure on the jejunal wall. As this rare complication of stent penetration occurred at a rate of 2.1% over 14 years, we considered it necessary to improve the stent insertion technique. Our revised approach involved inserting the pancreatic stent through the pancreatic duct and then advancing it an additional 5 cm distal into the jejunal lumen after passing it through the jejunal wall. The stent was then cut and left in place within the jejunal lumen. By advancing the stent further into the jejunal lumen, the risk of perpendicular contact and subsequent perforation of the jejunal wall was reduced. While this adjustment in the technique has successfully prevented stent-related jejunal perforations, complications such as stent migration into the jejunum and subsequent jejunitis have still been observed.

This case series emphasized the importance of careful consideration of the stent insertion techniques during PD, particularly in the placement angle and positioning of the pancreatic stent relative to the jejunal wall. While pancreatic stents play a crucial role in reducing POPFs, attention must be paid to their potential complications, including stent migrations and jejunal perforations. The described modification in the technique may serve as a useful strategy for surgeons to avoid such complications and improve patient outcomes; however, as complications related to stent migration can still occur, further investigation is warranted.

## Conclusions

Our cases highlight a rare but serious complication of a jejunal perforation due to internal pancreatic stents. Further research and a larger cohort of patients are necessary to validate the long-term efficacy and safety of this modified approach. Nevertheless, these cases highlight the critical need for vigilance and prompt management of complications associated with pancreatic stent migration in pancreatojejunostomy.

## References

[1] Shrikhande SV, Sivasanker M, Vollmer CM (2017). Pancreatic anastomosis after pancreatoduodenectomy: A position statement by the international study group of pancreatic surgery (ISGPS). Surgery.

[2] Bassi C, Falconi M, Salvia R, Mascetta G, Molinari E, Pederzoli P (2001). Management of complications after pancreaticoduodenectomy in a high volume centre: results on 150 consecutive patients. Dig Surg.

[3] Gouma DJ, van Geenen RC, van Gulik TM, de Haan RJ, de Wit LT, Busch OR, Obertop H (2000). Rates of complications and death after pancreaticoduodenectomy: risk factors and the impact of hospital volume. Ann Surg.

[4] Olakowski M, Grudzińska E, Mrowiec S (2020). Pancreaticojejunostomy-a review of modern techniques. Langenbecks Arch Surg.

[5] Motoi F, Egawa S, Rikiyama T, Katayose Y, Unno M (2012). Randomized clinical trial of external stent drainage of the pancreatic duct to reduce postoperative pancreatic fistula after pancreaticojejunostomy. Br J Surg.

[6] Kawai M, Yamaue H, Jang JY (2020). Propensity score-matched analysis of internal stent vs external stent for pancreatojejunostomy during pancreaticoduodenectomy: Japanese-Korean cooperative project. Pancreatology.

[7] Shin YC, Jang JY, Chang YR (2019). Comparison of long-term clinical outcomes of external and internal pancreatic stents in pancreaticoduodenectomy: randomized controlled study. HPB (Oxford).

[8] Jiang Y, Chen Q, Wang Z (2021). The prognostic value of external vs internal pancreatic duct stents in CR-POPF after pancreaticoduodenectomy: a systematic review and meta-analysis. J Invest Surg.

[9] Jang JY, Chang YR, Kim SW (2016). Randomized multicentre trial comparing external and internal pancreatic stenting during pancreaticoduodenectomy. Br J Surg.

[10] Kamoda Y, Fujino Y, Matsumoto I, Shinzeki M, Sakai T, Kuroda Y (2008). Usefulness of performing a pancreaticojejunostomy with an internal stent after a pancreatoduodenectomy. Surg Today.

[11] Hirono S, Kawai M, Yamashita Y (2018). Successful removal of an internal pancreatic stent that migrated into the bile duct using double-balloon enteroscopy after pancreaticoduodenectomy. Surg Today.

[12] Kadowaki S, Miura F, Amano H (2012). Whereabouts of an internal short stent placed across the pancreaticojejunostomy following pancreatoduodenectomy. J Hepatobiliary Pancreat Sci.

[13] Park SH, Kim JH, Noh SY (2015). Migration of internal pancreaticojejunostomy stents into the bile ducts in patients undergoing pancreatoduodenectomy. J Gastrointest Surg.

[14] Rezvani M, O'Moore PV, Pezzi CM (2007). Late pancreaticojejunostomy stent migration and hepatic abscess after Whipple procedure. J Surg Educ.

[15] Mari G, Costanzi A, Monzio N (2015). Small bowel perforation caused by pancreaticojejunal anastomotic stent migration after pancreaticoduodenectomy for periampullary carcinoma. JOP.

[16] Ortega PM, Zozaya-Larequi G, Arredondo J (2015). Distal migration of a transanastomotic pancreatic stent resulting in bowel perforation 19 years after pancreatoduodenectomy: report of a case. Surg Today.

[17] Bao L, Chen ZT, Huang JC, Li MX, Zhang LL, Wan DL, Lin SZ (2020). Small bowel perforation caused by pancreaticojejunal anastomotic stent migration after pancreaticoduodenectomy: A case report. Medicine (Baltimore).

